# MsGCN: a multi-stream graph convolutional network for multiband PLV graph fusion in EEG-based biometric identification

**DOI:** 10.3389/fncom.2026.1845003

**Published:** 2026-06-17

**Authors:** Wenli Tian, Jun Yang, Xiangyu Ju, Ming Li, Dewen Hu

**Affiliations:** 1 Northwest Institute of Nuclear Technology, Xi’an, Shaanxi, China; 2 College of Intelligence Science and Technology, National University of Defense Technology, Changsha, China

**Keywords:** EEG biometric identification, feature fusion, functional connectivity, MsGCN, multiband PLV

## Abstract

**Introduction:**

EEG-based biometric identification has attracted extensive attention due to its high security and uniqueness. Functional connectivity features derived from EEG exhibit strong individual specificity, yet existing methods do not fully leverage the complementary identity information contained in multiband functional connectivity features.

**Methods:**

This study proposes a multi-stream graph convolutional network (MsGCN) for EEG-based biometric identification by fusing graph representations derived from multiband phase-locking value (PLV) matrices. The model processes PLV matrices from multiple frequency bands through parallel GCN branches and performs end-to-end identification using fully connected layers. Experiments on the public PhysioNet Motor Movement/Imagery dataset evaluated the method under non-preprocessed conditions, cross-task settings, channel reduction, and different graph binarization thresholds.

**Results:**

MsGCN achieved 99.50% accuracy on preprocessed data and 98.12% on non-preprocessed data, showing numerically higher accuracy than the selected CNN and GCN baselines under the unified protocol. The model also showed improved robustness in cross-task identification, reduced-channel settings, and across a wide range of thresholds.

**Discussion:**

These results suggest that multiband PLV graph fusion can improve robustness to preprocessing conditions, task variation, channel reduction, and threshold selection under the evaluated dataset and experimental settings.

## 1 Introduction

Electroencephalograms (EEGs) reflect the brain’s electrical activity and contain subject-specific neural patterns. EEG biometrics are difficult to spoof and may remain relatively stable across different mental states and recording conditions. Accordingly, EEG-based identification may be particularly useful in applications requiring a high level of security (Mishra et al., 2024).

Physiological-signal-based biometrics have recently been investigated using multiple modalities, including respiratory-induced diaphragmatic electromyography (EMG) (Eraslan et al., 2023, 2025), otoacoustic emission (OAE) (Liu et al., 2023), electrocardiogram (ECG) (Ma et al., 2026), and EEG signals. For example, diaphragmatic EMG-based biometric systems exploit individual-specific respiratory muscle activation patterns, while stimulus-frequency OAE has been explored as an earprint modality with stability and resistance to replay or falsification attacks. ECG-based biometric studies have also investigated graph-based invariant representations to improve robustness against distribution shifts. Compared with these physiological modalities, EEG-based biometrics provide identity-related neural information and have potential advantages in non-replicability, inherent liveness detection, and resistance to coercion, although robustness across tasks, sessions, and recording conditions remains an important challenge (Tu et al., 2026).

The selection of EEG features to maximize differences among individuals is particularly important for EEG-based recognition. Commonly used EEG representations for biometric identification include power spectral density (PSD) (Meini et al., 2025), autoregression (AR) features (Guan et al., 2023), and discrete wavelet transform (DWT) (Wang et al., 2013). Additionally, some studies directly used raw EEG signals as features (Zhang et al., 2018). All of these features are single-channel features, and the connections among brain regions were not considered. For multichannel representations, the spatial covariance matrix carries the discriminative information of EEG signals and is widely used in EEG classification tasks (Ju et al., 2023). Common spatial pattern (CSP) is also one of the most popular methods for EEG feature extraction (Becerra et al., 2025). However, these representations are generally less effective for individual identification than connectivity-based features and are more commonly used in brain-computer interface (BCI) tasks. Recent EEG biometric studies have also explored dynamic temporal-pattern matching and cross-band fusion strategies. Ozturk et al. (2025) investigated EEG alpha-frequency authentication using t-SNE and dynamic time warping under resting-state and visual task-related conditions, showing the potential of task-related EEG dynamics for authentication. More recently, BrainprintNet introduced a multiscale cross-band fusion framework for EEG-based brainprint recognition and demonstrated improved generalization under cross-session and cross-task conditions (Tu et al., 2026).

Functional connectivity (FC) is characterized by enhanced individual specificity and stronger temporal stability than connectivity based on single-channel features and is considered promising for identification tasks. For biometric identification, effective EEG representations should not only maximize inter-subject separability but also maintain intra-subject repeatability across different task states or recording conditions. Therefore, evaluating whether identity-related connectivity patterns remain stable under task variation is important for assessing the practical reliability of EEG biometric systems. FC characterizes the dependence and coupling between different brain regions (Alonso and Vidaurre, 2023). Previous research on magnetic resonance imaging (MRI) has shown that FC networks exhibit a high degree of variability among individuals and act as individual “fingerprints” (Miranda-Dominguez et al., 2014). A variety of FCs, such as coherence (COH), correlation coefficient (CC), phase lag index (PLI), and phase locking value (PLV), have been used for identification tasks (Rizkallah, 2019; Wang et al., 2019). Fraschini et al. (2019) showed that PLI is more stable than time-domain features. Wang et al. (2020) investigated eight different FC metrics and found that the FC-based method is effective in improving the recognition rate and inter-state stability of the authentication system.

Recent studies have suggested that the discriminative capability of FC metrics for biometric identification is particularly pronounced for PLV and that PLV-based identification performance varies across frequency bands. Kong et al. (2019) used linear discriminant analysis (LDA) to classify extracted PLV features, and they found that EEGs are rhythmic and behave differently in different frequency bands and the accuracy of the β and γ bands is significantly higher than that of the α and θ bands. Wang et al. evaluated the individual specificity of EEG signals in different frequency bands based on a commonly used public dataset. In terms of overall trends, the high-frequency bands (β and γ) were most distinctively unique among individuals (Wang et al., 2021). In addition, the research of Fraschini et al. (2014) and Barra et al. (2017) also reached a similar conclusion.

Together, these studies suggest that FCs from different bands contain variable and complementary individual information (Gschwandtner et al., 2021). Thus, FCs from different bands can potentially be combined to improve the performance of identification systems (Ashenaei and Shirazi, 2025). However, simply combining different FCs into feature vectors may not improve accuracy and increases the computational overhead in classification (Pan et al., 2021; Yu, 2020). However, how to effectively integrate multiband FC information while preserving graph structure remains an open challenge in EEG biometric identification.

In our preliminary conference work, we explored the use of multiband PLV-derived graphs for EEG-based identification and introduced an initial multi-stream GCN framework for fusing frequency-specific connectivity information (Tian et al., 2023). The present study extends that preliminary work both algorithmically and experimentally. Algorithmically, the graph representation and network structure were optimized by using node-strength-based input features derived from the original weighted PLV matrices. This design reduces one graph convolution operation while preserving first-order connectivity-strength information, thereby providing a more compact implementation of the multi-stream graph-fusion strategy. In addition, the proposed method performs representation-level fusion of frequency-specific PLV-derived graphs through independent GCN branches, rather than directly concatenating raw connectivity matrices or using a single graph representation.

Experimentally, the present work expands the evaluation from the EO/EC baseline tasks considered in the preliminary study to four task conditions, including EO, EC, motor execution, and motor imagery. Mixed-task and cross-task experiments were added to examine whether identity-related PLV graph representations remain stable across task states. Additional robustness analyses were also conducted under non-preprocessed data, reduced-channel settings, and different PLV binarization thresholds.

The main contributions of this study are as follows:

(1) we present an optimized multi-stream GCN architecture for representation-level fusion of multiband PLV-derived graphs;

(2) we introduce a node-strength-based graph representation that reduces one graph convolution operation while preserving first-order connectivity-strength information;

(3) we evaluate the model under non-preprocessed, mixed-task, cross-task, and reduced-channel settings to examine robustness across different experimental conditions;

(4) we analyze the sensitivity of the proposed model to PLV graph binarization thresholds.

The remainder of this paper is organized as follows: Section “2 Materials and methods” introduces the details of the method and the proposed MsGCN model, Section “3 Results” presents the evaluation results of the proposed method, and the results are discussed, and Section “4 Conclusion” concludes the findings of this research.

## 2 Materials and methods

### 2.1 Dataset

The proposed model was tested with the PhysioNet EEG Motor Movement/Imagery dataset (Schalk et al., 2004; Goldberger et al., 2000). As one of the most widely used publicly available datasets in the field of EEG, this dataset allows the results of the proposed model to be compared with those of other models. This dataset contains more than 1,500 one-minute and two-minute EEG records collected from 109 subjects. Each subject was involved in 14 experiments: two one-minute baseline experiments, namely, eye opening (EO) and eye closing (EC), and three two-minute experiments of four kinds involving motor or imagination tasks. EEG signals were collected with 64 conductive electrodes (excluding electrodes A1 and A2) in the international 10–10 system, and the sampling rate was 160 Hz.

The experiment was mainly based on the data from four tasks: EO, EC, motor execution (opening/closing the left or right fist; denoted T1) and motor imagery (imagining opening/closing the left or right fist; denoted T2).

### 2.2 EEG preprocessing

The EEG recordings were segmented using a sliding-window strategy. To avoid potential data leakage caused by overlapping windows, the train/test split was performed before window segmentation. Specifically, in each fold of the fivefold cross-validation, continuous EEG time indices were first divided into training and testing partitions. Preprocessing operations, sliding-window segmentation, normalization, and PLV-based graph construction were then performed within the corresponding training and testing partitions. Therefore, overlapping windows derived from the same continuous EEG segment were not randomly assigned to both the training and testing sets.

Both preprocessed and non-preprocessed data were used to evaluate the proposed model. For the preprocessed data, independent component analysis (ICA) was applied to the training data and then used to transform the corresponding testing data. For the non-preprocessed data, no ICA-based artifact removal was applied; only the same train/test partitioning and sliding-window segmentation procedure was used.

### 2.3 Feature extraction

A feature extraction process was used to calculate PLVs in different frequency bands, which were then used as inputs to the subsequent modules. PLV quantifies the consistency of phase differences between two signals across time and is defined as:


$$P\,L\,V=\left|n^{-1}\,\overset{n}{\underset{t=1}{\sum }}e^{i\,\left(\phi_{x\,t}-\phi_{y\,t}\right)}\right|$$

where *n* denotes the number of time points and *ϕ*_*xt*_ and *ϕ*_*yt*_ represent the phase angles of signals *x* and *y* at time point *t*, respectively. The range of PLV is [0, 1], and the larger the value is, the stronger the phase synchronization between the two signals.

When calculating PLV, it is usually necessary to divide the frequency bands. The traditional frequency band division methods are the δ, θ, α, β, and γ methods. Previous EEG biometric studies have reported that frequency-band-specific connectivity features show different discriminative abilities (Barra et al., 2017; Fraschini et al., 2014; Kong et al., 2019; Tu et al., 2026; Wang et al., 2021). Recent cross-band EEG brainprint studies have also suggested that user identity is related to specific EEG frequency subbands and channels (Tu et al., 2026). To reduce the imbalance in bandwidth among conventional EEG bands, we grouped δ, θ, and α into a broader “*other*” band (1–13 Hz), while retaining β (14–30 Hz) and γ (31–44 Hz) as separate bands. This grouping was adopted for two reasons. First, previous EEG biometric studies have suggested that β- and γ-band connectivity features generally contain stronger subject-specific information than lower-frequency bands (Tian et al., 2023; Kong et al., 2019; Wang et al., 2021). Therefore, β and γ were kept as independent streams in the proposed MsGCN. Second, combining δ, θ, and α into one low-frequency composite band produces a more balanced multiband representation and reduces the number of input streams and model parameters. Thus, the three-band setting was used as a practical compromise between preserving discriminative high-frequency information and maintaining a compact model structure.

### 2.4 Graph construction

**FIGURE 1:**
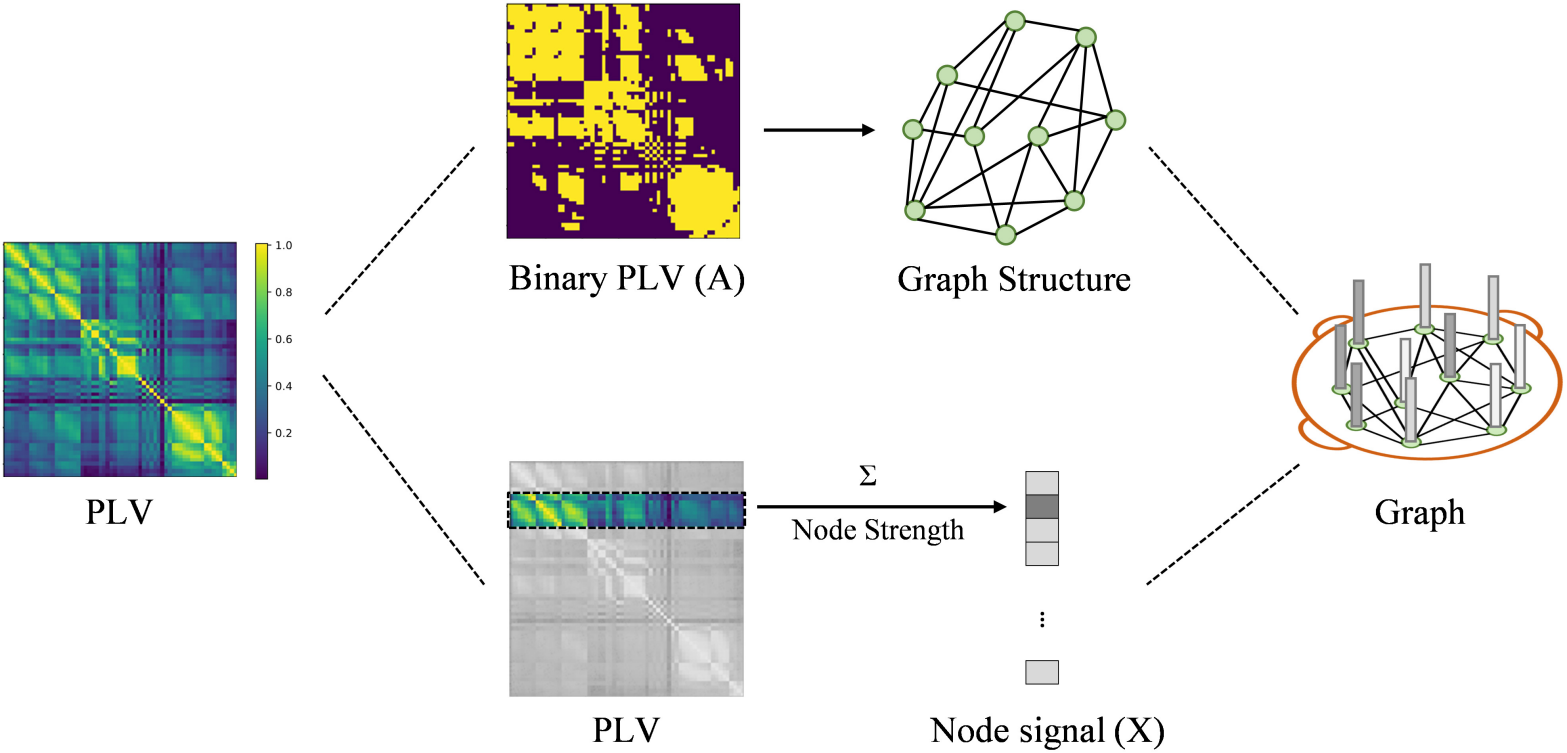
Graph representation based on PLVs, the edge of the graph is constructed based on a binary PLV, and the node strength is calculated as a node feature.

The graph representation process based on PLV matrices is illustrated in Figure 1. Because the PLV matrix defines a dense weighted graph, thresholding was applied to obtain a sparse binary adjacency matrix. PLV values are naturally bounded between 0 and 1, where larger values indicate stronger phase synchronization. In this study, the PLV threshold was treated as a fixed hyperparameter and applied consistently to all PLV matrices under the same experimental setting, rather than being calculated separately for each sample or estimated from the global data distribution. Specifically, for each PLV matrix, elements greater than the selected threshold were set to 1, whereas the remaining elements were set to 0. In the initial experiments, the threshold was empirically set to 0.75. To further evaluate the influence of this choice, threshold sensitivity was analyzed by varying the fixed threshold from 0 to 1. The threshold directly influences the number of retained edges and therefore affects classification performance.

In addition, node features are required for graph construction, and node strength was used as the node feature in this study. The node-strength value was first calculated for each EEG channel and then used as the initial node information for constructing the node-feature representation of each frequency-specific graph. Specifically, for each frequency band, let $P^{\left(b\right)}=\left[P_{i\,j}^{\left(b\right)}\right]\in {\mathbb{R}}^{\text{N}\times \text{N}}$ denote the original weighted PLV matrix of a given EEG window before binarization, where N is the number of EEG channels and *b* denotes the frequency band. The node strength of channel *i* was calculated as the sum of its weighted PLV connections with all other channels:


$$s_{i}^{\left(b\right)}=\overset{N}{\underset{j=1,j\neq i}{\sum }}P_{i\,j}^{\left(b\right)}$$

Therefore, each node-strength value represents the overall weighted phase-synchronization strength between one EEG channel and the remaining channels. The node-strength value of each channel was used as the initial node descriptor for constructing the node-feature representation of each frequency-specific graph. In the implemented network, the input feature matrix of each stream was represented as X_(b)_ ∈ ℝ^64 × 32^, where 64 denotes the number of EEG channels and 32 denotes the node-feature dimension used in the GCN branch. This feature dimension was kept consistent with the hidden feature dimension of the single-stream GCN baseline. In our graph representation, the original PLV matrix was used to compute node-strength-based features, whereas the binarized PLV matrix was used as the graph adjacency matrix.

This design can be regarded as an optimized form of using an all-one node-signal vector in the first GCN layer (Tian et al., 2023), because multiplying the PLV matrix by an all-one vector is equivalent to summing the PLV connections of each node. Therefore, using node strength as the initial node feature reduces one graph convolution operation while preserving the first-order connectivity-strength information.

In this study, the term “fusion” refers to representation-level fusion of frequency-specific PLV-derived graphs rather than direct fusion of raw functional connectivity matrices. Specifically, a PLV matrix is first computed for each frequency band and then converted into a graph representation with a binary adjacency matrix and node-strength features. These band-specific graphs are processed by independent GCN branches, and the learned graph representations are concatenated and fused in the subsequent fully connected layers.

### 2.5 MsGCN model architecture

**FIGURE 2:**
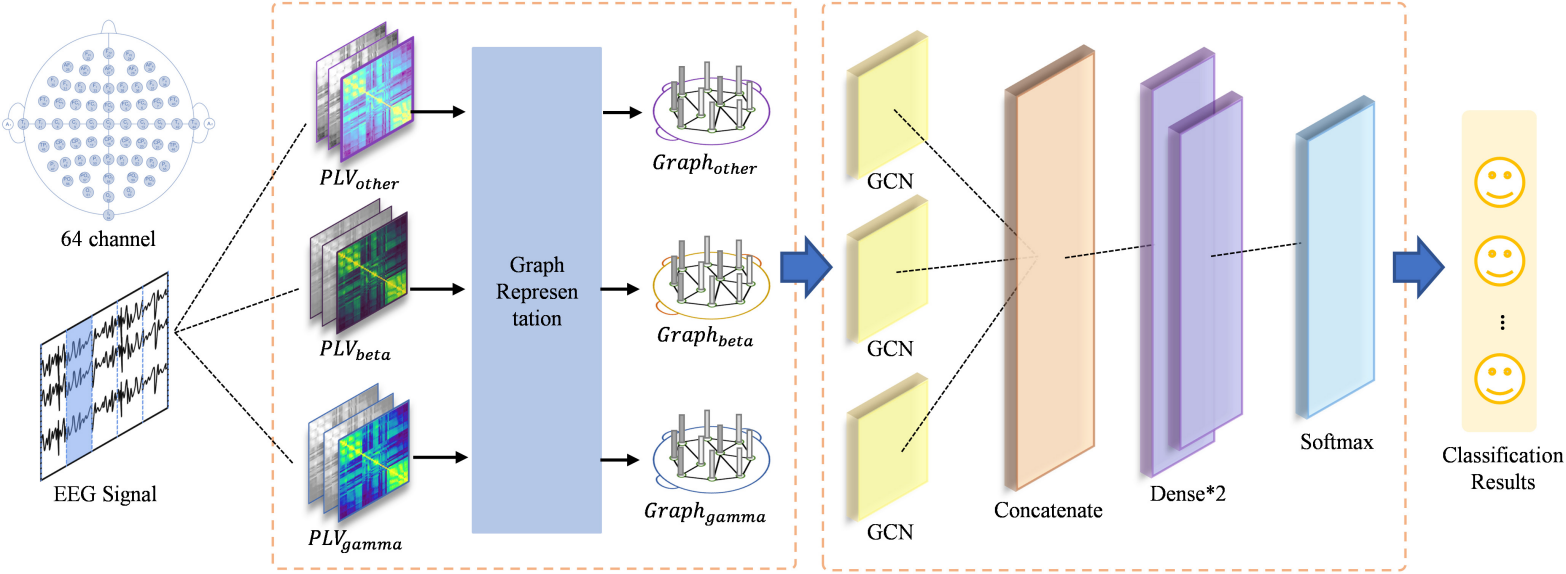
MsGCN framework for EEG identification.

The general framework of the MsGCN model for EEG identification is shown in Figure 2, and the original EEG signals are divided with a sliding window. For each division, the PLVs of the *other*, β and γ frequency bands are obtained, and a graph representation is established based on these PLVs to obtain multiband graph signals. Then, the MsGCN model is applied for classification.

MsGCN is a network mainly composed of a GCN layer and a fully connected layer. The network has three inputs, and multiband graph signals are obtained from graph representation. First, three parallel GCN branches with identical architecture are used to aggregate the PLV information from different channels. A single layer GCN is defined as:


$$X^{1}=\sigma_{t\,a\,n\,h}\left(A,X^{0}|W^{1},b^{1}\right)W^{1}\in {\mathbb{R}}^{32\times 8},b^{1}\in {\mathbb{R}}^{8}$$

where *X* is a node feature, *A* is the adjacency matrix, σ is the activation function, and *W* and *b* are the weight and the bias, respectively. Here, *X*^0^ denotes the node-strength-based initial input feature matrix, *A* denotes the binary PLV adjacency matrix, and *tanh* is selected as the activation function.

After the GCN layer, a concatenation layer is used to aggregate multiband PLV features. Different from direct matrix-level fusion or simple feature-vector concatenation, this architecture first learns band-specific graph representations through independent GCN branches and then fuses the learned representations in the subsequent fully connected layers. This design allows each frequency band to maintain its own graph topology and trainable parameters before cross-band integration. Then, two fully connected layers are used to learn discriminative representations from the fused multiband features. In the output layer, Softmax is used for classification.

### 2.6 Experimental implementation details

The model is implemented based on the Python 3.7 and TensorFlow 2.3.1 framework. NVIDIA GeForce RTX 2080Ti is used for model training.

### 2.7 Baseline models

In this section, a CNN (Wang et al., 2019) and a GCN (Behrouzi and Hatzinakos, 2021) are introduced as the baseline models. Both architectures are classical, widely recognized, and representative benchmarks in EEG-based biometric identification tasks, and their well-documented network designs ensure high reproducibility in relevant experimental validations. Despite the evolving landscape of relevant research, these two models remain the most commonly adopted baselines for evaluating the performance of novel EEG identification frameworks due to the limited emergence of new dedicated models for this specific dataset and task in recent years. Table 1 describes the detailed construction of the two baseline models and the proposed MsGCN. All models are trained for 109-class classification corresponding to 109 subjects in the dataset. In this study, the experiments were formulated as a closed-set multi-class identification task, in which each EEG sample was assigned to one of 109 enrolled subjects. Therefore, classification accuracy was adopted as the primary evaluation metric. Verification-oriented metrics, such as ROC/AUC, false acceptance rate (FAR), false rejection rate (FRR), and equal error rate (EER), require a dedicated score-level authentication protocol involving genuine and impostor comparisons and were not evaluated in the present closed-set identification setting. Additionally, fivefold cross-validation was conducted for experimental evaluation, with approximately 20% of the continuous EEG data designated as the testing partition in each fold.

**TABLE 1:** Model construction for the baseline methods and the proposed method.

Model	Layer	Output	Kernel
CNN (Wang et al., 2019)	Input	64 × 64	–
	Convolution	63 × 63 × 32	4 × 4 (32)
	Max-pooling	31 × 31 × 32	2 × 2
	Convolution	30 × 30 × 32	4 × 4 (32)
	Max-pooling	15 × 15 × 32	2 × 2
	Flatten	7,200	–
	Dense	128	–
	Output (softmax)	109	–
GCN (Behrouzi and Hatzinakos, 2021)	Input	64 × 1	–
	GCN layer	64 × 32	1 × 32
	GCN layer	64 × 8	32 × 8
	Flatten	512	–
	Dense	109	–
	Output (softmax)	109	–
MsGCN	Input*	64 × 32	–
	GCN layer*	64 × 8	32 × 8
	Concatenate	192 × 8	–
	Flatten	1,536	–
	Dense	218	–
	Output (softmax)	109	–

*Three-stream input, followed by a three-stream GCN layer. The structure of the three streams is the same. Only the parameters of one stream are shown here.

It should be noted that the comparison among CNN, GCN, and MsGCN is not intended to isolate only the effect of network depth, parameter count, or identical input tensor size, because these models use inherently different input representations and model architectures. The CNN baseline treats the PLV matrix as a two-dimensional image-like input, the GCN baseline processes a single PLV-derived graph representation, and the proposed MsGCN processes multiple frequency-specific PLV-derived graphs through parallel streams. Therefore, the comparison is designed to evaluate whether the proposed multistream graph representation is more effective than representative matrix-based and single-graph modeling strategies. To ensure a fair experimental protocol, all models were trained and tested using the same EEG recordings, train/test partitions, preprocessing settings, sliding-window segmentation strategy, and fivefold cross-validation procedure.

## 3 Results

### 3.1 Identification accuracy on preprocessed/non-preprocessed data

Before presenting the results under our unified experimental protocol, we first summarize reported accuracies of representative EEG biometric identification methods that used the PhysioNet EEG Motor Movement/Imagery dataset or closely related PhysioNet-based settings. It should be noted that these studies may differ in preprocessing procedures, feature extraction methods, task selection, segmentation strategies, and train/test protocols. Therefore, the comparison in Table 2 should be interpreted as a reference comparison rather than a strictly controlled head-to-head evaluation. The controlled comparison in this study is conducted among CNN, GCN, and the proposed MsGCN under the same data partitions and evaluation protocol.

**TABLE 2:** Reported results of representative EEG biometric methods on selected PhysioNet-based settings (preprocessed data).

Method	Feature	ACC (EO)	ACC (EC)
Distance-based classifier (Barra et al., 2017)	ECG + EEG (β) fusion data	97.51	–
SVM (Kaur and Singh, 2017)	DWT and statistical features	96.88	96.02
RF (Kaur and Singh, 2017)	DWT and statistical features	95.78	93.21
CNN (Ma et al., 2015)	Raw EEG data	88.00	–
CNN (Wang et al., 2019)	All-band PLVs	97.86	96.72
GCN (Behrouzi and Hatzinakos, 2021)	All-band PLVs	99.21	98.62
Mahalanobis distance classifier (Yahyaei and Ozkurt, 2022)	Mean Curve Length (MCL)	**99.4%**	**98.8%**
MsGCN (ours)	*other* + β + γ PLVs	99.50	**99.12**

EO and EC denote the eye-open and eye-closed baseline tasks in the PhysioNet EEG Motor Movement/Imagery dataset. The settings of the compared methods follow the corresponding original studies and may differ in preprocessing, segmentation, and validation protocols. Bold values indicate the best performance under each corresponding condition; for accuracy, this denotes the highest value, and for accuracy drop, this denotes the smallest value.

The accuracy of our proposed MsGCN model is compared with that of several existing models and the results are shown in Table 2. As can be seen from Table 2, almost all methods achieve more than 90% accuracy. For the EO and EC tasks, the proposed MsGCN achieved accuracies above 99%. Although the results in Table 2 are not directly comparable in a strictly controlled sense because of differences in experimental protocols, they indicate that the proposed method achieves competitive performance among representative PhysioNet-based EEG biometric studies.

However, high accuracy on preprocessed data alone is insufficient for a practical biometric system. We therefore evaluated performance on non-preprocessed data. We evaluated the classification accuracy of three models, the CNN, GCN and MsGCN, based on four tasks and compared the results in these cases with those obtained with preprocessed data. The results are shown in Table 3.

**TABLE 3:** Identification accuracy on preprocessed/non-preprocessed data.

Task	Method	Preprocessed	Non-preprocessed	Accuracy drop (preprocessed—non-preprocessed)
EO	CNN	97.86	89.72	8.14
	GCN	99.21	95.15	4.06
	MsGCN	**99.50**	**98.12**	**1.38**
EC	CNN	96.72	86.33	10.39
	GCN	98.62	92.19	6.43
	MsGCN	**99.12**	**96.45**	**2.67**
T1	CNN	97.57	88.62	8.95
	GCN	99.22	94.40	4.82
	MsGCN	**99.55**	**97.60**	**1.95**
T2	CNN	97.70	89.13	8.57
	GCN	99.31	94.41	4.90
	MsGCN	**99.60**	**97.65**	**1.95**

Bold values indicate the best performance under each corresponding condition; for accuracy, this denotes the highest value, and for accuracy drop, this denotes the smallest value.

The proposed MsGCN model achieves the highest accuracy for the non-preprocessed data, with values of 98.12% (EO), 96.45% (EC), 97.60% (T1) and 97.65% (T2). Compared with that obtained with the preprocessed data, the accuracy of the data without preprocessing is reduced to varying degrees. However, compared with that of the other two methods, the accuracy of the proposed MsGCN method decreases less. These findings suggest that MsGCN is more robust to noise and artifacts and can better preserve discriminative identity-related information.

It should be noted that some accuracy differences between MsGCN and the selected baselines on preprocessed data were small. Therefore, these differences should be interpreted cautiously as numerical performance differences under the present cross-validation protocol, rather than as evidence of statistical significance.

### 3.2 Ablation study on single-band and multiband PLV graphs

To evaluate the contribution of the multi-stream architecture, we compared the proposed MsGCN with single-band GCN models using PLV graphs from the other, β, and γ bands. The results are shown in Table 4. The proposed MsGCN achieved the highest accuracy across all four tasks, with accuracies of 99.50, 99.12, 99.55, and 99.60% on EO, EC, T1, and T2, respectively. In contrast, the single-band GCN models showed slightly lower performance. These findings suggest that although β- and γ-band PLV graphs contain strong subject-specific information, the three-stream fusion of PLV graphs from the other, β-, and γ-bands provides complementary identity-related information and improves the final identification performance.

**TABLE 4:** Single-band and multiband ablation results.

Data band	ACC (%)			
	EO	EC	T1	T2
*Other*	99.26	98.60	99.23	99.38
β	99.15	98.65	99.24	99.32
γ	99.26	98.51	99.23	99.32
*Other* + β + γ	**99.50**	**99.12**	**99.55**	**99.60**

Bold values indicate the best performance under each corresponding condition; for accuracy, this denotes the highest value, and for accuracy drop, this denotes the smallest value.

It should be noted that the improvement over single-band GCNs is moderate on preprocessed data, because all single-band PLV graphs already achieve high accuracy on this dataset. However, the consistent improvement across all tasks supports the effectiveness of the proposed multiband fusion strategy. In addition, the robustness analyses on non-preprocessed data, mixed-task data, cross-task data, and reduced-channel settings further indicate that MsGCN provides a more stable representation for EEG-based biometric identification.

Previous EEG biometric studies have reported that β- and γ-band connectivity features are highly discriminative for individual identification (Kong et al., 2019; Wang et al., 2021). In addition, recent cross-band brainprint recognition studies have shown that combining information from multiple frequency subbands can improve EEG-based identification, especially under challenging cross-task or cross-session settings (Tu et al., 2026). In this study, the single-band ablation results also show that β- and γ-band PLV graphs achieve strong performance. However, the proposed *other* + β + γ MsGCN still achieves consistently higher accuracy than each single-band GCN, suggesting that the low-frequency composite band may provide complementary identity-related information beyond β and γ alone. Although additional two-band combinations, such as β + γ, were not exhaustively evaluated in the present study, the current results support the effectiveness of the proposed three-stream multiband design under the evaluated setting.

### 3.3 Mixed-task and cross-task performance

An effective EEG-based authentication model should be able to accurately identify users by their brain waves, regardless of the task state. This requirement is closely related to intra-subject repeatability: identity-related features from the same subject should remain sufficiently consistent when the subject performs different tasks, while remaining distinguishable from those of other subjects. To verify the ability of the proposed model to achieve this type of identification, a dataset of four tasks (EO\EC\T1\T2) is used. The amount of data available for the four tasks is equal. We tested our model with mixed-task data and compared it with two baseline methods. For mixed-task performance, the training and test sets are randomly sampled from the data containing all tasks. The results are shown in Table 5.

**TABLE 5:** Identification accuracy of different methods based on mixed-task data.

Data	Method	Preprocessed	Non-preprocessed
Mixed-task data (EO\EC\T1\T2)	CNN	96.45	83.24
	GCN	97.81	89.66
	MsGCN	**99.40**	**95.42**

Bold values indicate the best performance under each corresponding condition; for accuracy, this denotes the highest value, and for accuracy drop, this denotes the smallest value.

Table 5 shows that the proposed MsGCN model achieves the highest accuracy at 99.40% on preprocessed data and 95.42% on non-preprocessed data. The GCN ranks second, and the CNN yields the lowest accuracy. These results suggest that the proposed MsGCN model can capture identity-related information from multitask EEG data. To further verify this finding, we designed a cross-task experiment to determine whether the proposed model can process EEG signals for unknown tasks.

**FIGURE 3:**
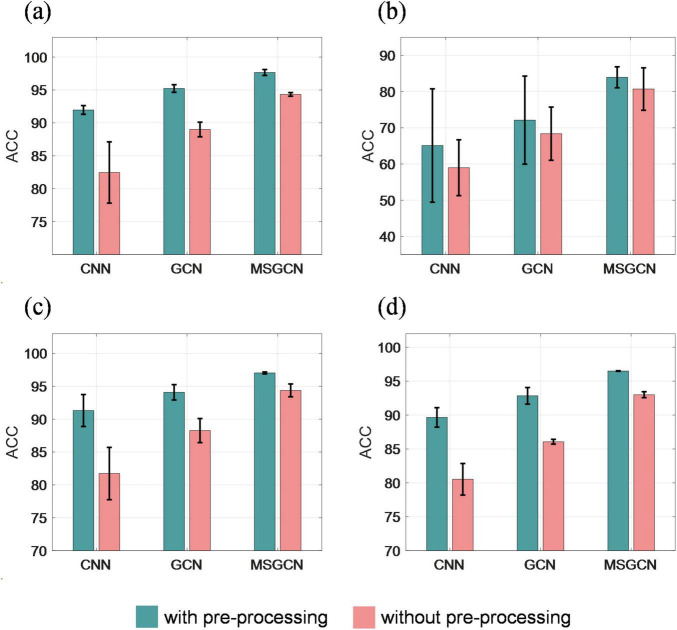
Classification accuracy of three models based on cross-task datasets. Under the condition of processing unknown task data, the proposed MsGCN model achieves the highest accuracy when four tasks are considered in the test set. The GCN ranks second, and the CNN yields the lowest accuracy. **(a)** EO, **(b)** EC, **(c)** T1, and **(d)** T2.

For Cross-Task Performance, data from four states (EO, EC, T1, and T2) are included in the test set, and training data used are from three other tasks. The accuracies of the proposed MsGCN model and the two baseline methods are compared. The results are shown in Figure 3. Bars indicate mean values across repeated experiments, and error bars represent the standard deviation (SD).

Figure 3 shows that the proposed MsGCN model achieves the highest accuracy when data from four kinds of tasks are included in the test set for processing unknown task data. The GCN ranks second, and the CNN yields the lowest accuracy. Notably, the DA-SCNN model (Alattabi et al., 2025) was recently evaluated on unseen session data of the same dataset to verify cross-session identification effectiveness, achieving an accuracy of 95.7% with the Delta-band Baseline. In contrast, our MsGCN model reaches a higher accuracy of 97.65% (preprocessed) and 94.32% (unpreprocessed) in cross-task scenarios. Although the experimental settings differ and direct comparison should therefore be interpreted cautiously, the reported performance of MsGCN in the cross-task setting is competitive with recently reported results on related generalization tasks.

At the same time, it can be seen from Figure 3 that the accuracy when the EC task data are used is lower than that when data from the other tasks are used in the test set. This may be because when an individual is resting with their eyes closed, they are asked not to perform any tasks, resulting in diverse and unpredictable human brain activities.

In terms of the influence of preprocessing, the accuracy achieved with non-preprocessed data is lower than that obtained with preprocessed data. However, the accuracy of the proposed MsGCN model decreases less, which suggests that the model is more tolerant to artifacts and better able to preserve identity-related information across tasks.

### 3.4 Robustness to channel reduction

Table 2 shows that all three models can achieve high accuracy by using 64 channels of data. However, increasing the number of EEG channels increases the cost of establishing an identity system. In practical applications, it is costly to arrange 64 channels for brain monitoring, and it may be necessary to balance identification performance and acquisition/computational cost (Aljalal et al., 2024). Therefore, we evaluated the sensitivity of the proposed MsGCN model to the number of channels. Specifically, we assessed the changes in the accuracy of the three models when the number of channels was decreased.

**FIGURE 4:**
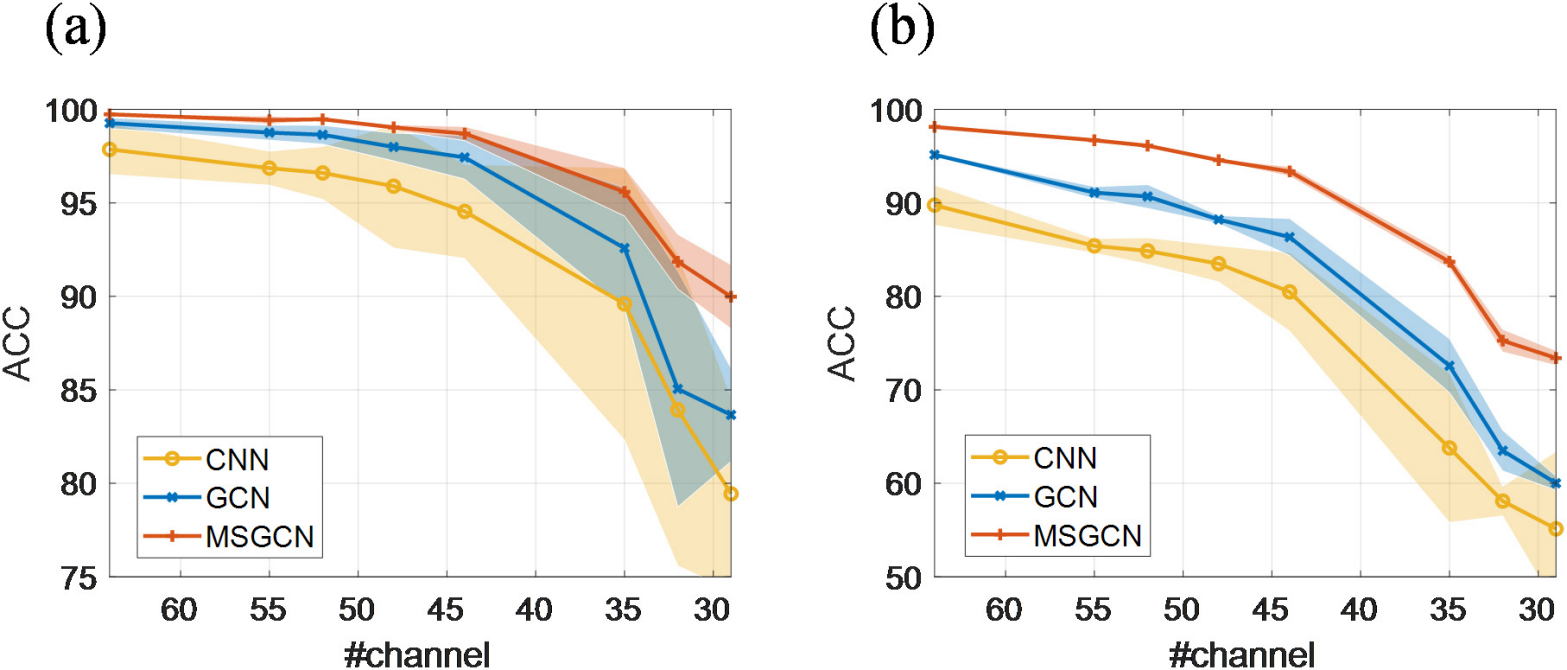
Classification accuracy of the three models based on different channel reduction degrees: **(a)** EO and **(b)** EC.

We extracted a number of different channels from the 64 channels of the brain according to the spatial distribution of the channels, used only the extracted channel data for classification, and compared the classification accuracy of the three models. Since typical channel selection methods may affect EEG classification accuracy (Alyasseri et al., 2020), we chose spatially evenly dispersed channels to reduce the risk of disproportionate influence of channels located in a particular brain region. The results are shown in Figure 4. Dots indicate mean values across repeated experiments, and error bars represent the standard deviation (SD).

Figure 4 shows that as the number of channels decreases, the accuracy of all three models decreases. However, the proposed MsGCN model displays the slowest decline in accuracy, with higher accuracy in the evaluated channel-reduction settings (i.e., for any number of channels). When the number of channels is reduced to half of the original number (32 channels), the accuracy of the MsGCN model still reaches more than 90% for the EO task and is nearly 10% higher than that of the GCN for the EC task.

As a network feature, a PLV is more sensitive to the number of channels than to single-channel EEG features (e.g., PSD). When the number of channels is reduced to half of the original number, the amount of data that a PLV can process is reduced to approximately 1/4 of the original amount, resulting in an extensive negative impact on classification. However, compared with the CNN and GCN models, which also use PLVs as features, the MsGCN yields a higher and more stable correct classification rate, indicating that the proposed model is less sensitive to channel reduction under the evaluated setting. This result suggests that multiband PLV graph fusion may be useful for future studies on reduced-channel EEG biometric systems, although additional validation is needed before drawing practical deployment conclusions.

### 3.5 Threshold sensitivity analysis

**FIGURE 5:**
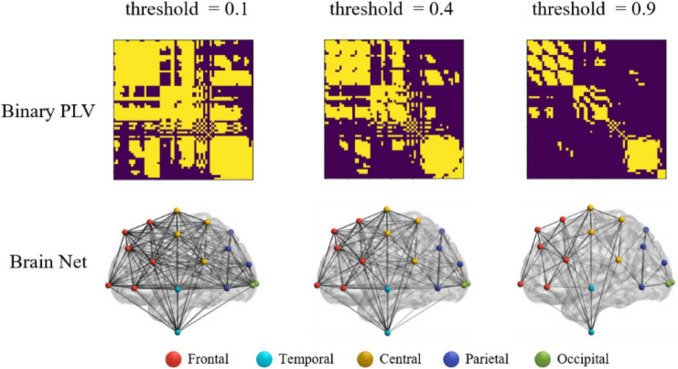
Influence of the threshold value of PLV binarization.

Here, we propose the concept of a threshold sensitivity in the model for PLV binarization. PLVs describe the phase relationships in the frequency domain among different EEG electrodes, and the selection of the threshold for PLV binarization directly influences the number of interconnected edges in PLVs, thus affecting the classification result. Figure 5 shows the influence of the threshold value of PLV binarization on the proposed graph structure.

In Figure 5, from left to right, as the threshold gradually increases, the number of interconnected edges gradually decreases, and the network tends to be simple. If the threshold value is too small, the graph structure will be overly complicated and may contain noise, leading to reduced accuracy; however, if the threshold value is too large, the graph structure will be too sparse, and more GCN layers will need to be added to aggregate the adjacent node information. Therefore, for a certain model, the most suitable threshold is within a given range. The larger the range is, that is, the lower the threshold sensitivity of the model, the greater the number of binary thresholds that can be used and the easier the network design. The smaller the range is, that is, the higher the sensitivity of the model to threshold, the smaller the number of binary thresholds that can be used. In this case, the risk of producing low-accuracy results due to improper threshold selection is comparatively high. In practice, model training usually does not need to be limited to an optimal threshold, and it is a time-consuming and laborious process to sequentially test thresholds for a given model. Therefore, if the sensitivity of a model to the threshold is low, the number of reasonable thresholds will be high, resulting in a reduced debugging cost.

**FIGURE 6:**
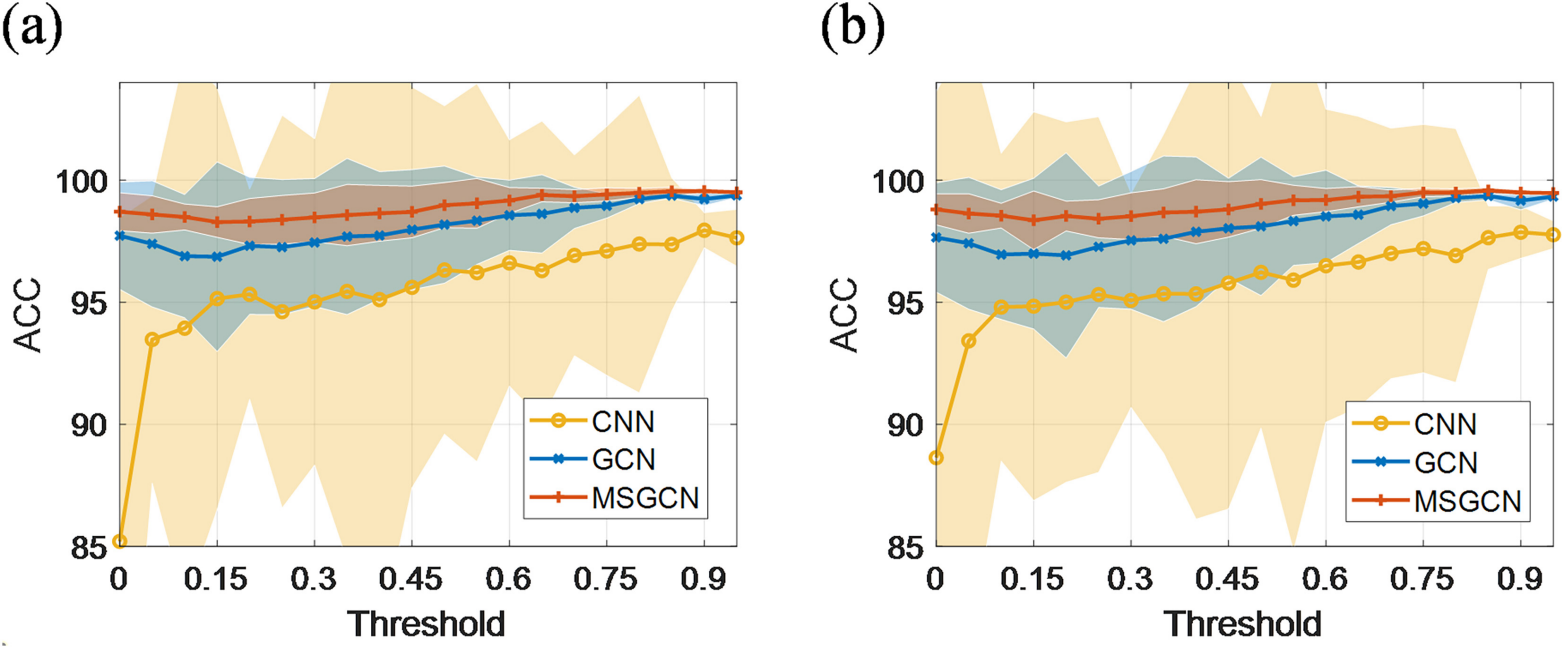
Changes in the accuracy of the three models with the threshold value for the EO task: **(a)** non-preprocessed data and **(b)** preprocessed data. The proposed MsGCN and GCN models maintain high accuracy under almost all threshold conditions, and the accuracy of the CNN is low at low thresholds (<0.15).

To test the sensitivity of the three models to different thresholds, we use the data from the EO task to binarize PLVs with thresholds that vary from 0 to 1 and compare the accuracy of the three models. The results are shown in Figure 6.

Figure 6 illustrates that the MsGCN and GCN models maintain high accuracy at almost all thresholds, while the accuracy of the CNN is low when the threshold is small (< 0.15). A small threshold is associated with a complex graph structure, which suggests that the CNN may have difficulty removing the noise from PLVs when dealing with a complex graph structure, resulting in unsatisfactory performance. The GCN and the proposed MsGCN performed well across a broad range of thresholds, suggesting that graph-convolution-based classifiers are well suited for processing graph-structured connectivity data. Considering the threshold selection process in practical applications, the GCN and the proposed MsGCN model display lower threshold sensitivity under the evaluated setting. This finding suggests that graph-convolution-based classifiers may reduce the dependence on precise threshold tuning, although deployment-oriented conclusions require further validation on additional datasets and recording conditions.

## 4 Conclusion

Identification technology based on EEG functional connectivity features has gained increasing research attention. The existing research has shown that PLV classification ability varies in different frequency bands, which implies that PLVs in different frequency bands are associated with different identity information. However, models and related methods that can be used to comprehensively utilize PLV characteristics in different frequency bands are lacking. In this paper, an MsGCN network structure that uses the PLVs of different frequency bands at the same time is proposed. Multiband PLV features are extracted through multiple GCN layers with the same structure, and then they are fused in a later network. The useful features among the multiband features are selected in a fully connected layer. We evaluated the proposed MsGCN model on a widely used public dataset and compared it with representative reported methods and selected CNN/GCN baselines. The results show that the proposed model achieved identification accuracies of 99.50% and 98.12% for preprocessed and non-preprocessed data, respectively, and showed numerically higher accuracy than the selected CNN and GCN baselines under the unified protocol. Additionally, the proposed method was evaluated in multitask settings, achieving over 99.40% accuracy on mixed-task data and 97.65% accuracy on cross-task data. These results suggest that the proposed MsGCN can provide competitive and robust performance across the evaluated experimental settings, although small numerical differences should be interpreted cautiously without fold-level statistical testing. Finally, we discuss the sensitivity of the proposed model to changes in the channel number and binarization threshold and verify that the MsGCN model is insensitive to decreases in the channel number.

Overall, the results indicate that multiband PLV graph fusion with a multi-stream GCN can improve robustness to preprocessing conditions, task variation, and channel reduction under the evaluated experimental settings.

This study has several limitations. First, the evaluation is restricted to a single public dataset. Therefore, the present results should be interpreted within the evaluated PhysioNet-based experimental setting, and cross-dataset validation on other EEG biometric datasets is needed before making general claims about real-world deployment. Second, although the mixed-task and cross-task experiments provide preliminary evidence of intra-subject repeatability across task states, cross-session and long-term stability were not tested in this study. These factors are critical for deployment-oriented EEG biometric systems. Future studies may also incorporate invariant representation learning or cross-session/cross-task adaptation strategies to improve the generalization ability of EEG biometric models under real-world distribution shifts (Ma et al., 2026; Tu et al., 2026). Third, this study focused on closed-set multi-class identification, and verification-oriented metrics such as ROC/AUC, FAR, FRR, and EER were not evaluated because a dedicated score-level authentication protocol was not implemented. Future work should evaluate the proposed method under both identification and verification settings. Fourth, the frequency band grouping and graph binarization strategy are empirical. Although the proposed *other* + β + γ setting was shown to outperform the single-band settings, additional band combinations, such as β + γ or other two-band configurations, were not exhaustively evaluated. Automated or data-driven band selection methods should be explored in future work.

## Data Availability

Publicly available datasets were analyzed in this study. This data can be found here: https://physionet.org/content/eegmmidb/1.0.0/.
